# Water exercises and quality of life during pregnancy

**DOI:** 10.1186/1742-4755-8-14

**Published:** 2011-05-16

**Authors:** Ana L Vallim, Maria J Osis, José G Cecatti, Érica P Baciuk, Carla Silveira, Sérgio R Cavalcante

**Affiliations:** 1 Department of Obstetrics and Gynecology, School of Medical Sciences, University of Campinas (UNICAMP), São Paulo, Brazil; 2 Centre for Research in Reproductive Health of Campinas (CEMICAMP), São Paulo, Brazil

## Abstract

**Background:**

In Brazil, concern with the quality of life of pregnant women is one of the points emphasized in the Program for the Humanization of Prenatal Care and Childbirth launched in 2000. However, there are few references in the literature on the role of either land or water-based physical exercise on women's quality of life during pregnancy. The purpose of this study was to evaluate the effects of a physical exercise program of water aerobics on the quality of life (QOL) of sedentary pregnant women.

**Methods:**

A comparative observational study involving sedentary low-risk pregnant women bearing a single fetus with gestational age less than 20 weeks at the time of admission to the study, who were receiving antenatal care at a public health service. One group of 35 women was given routine antenatal care, while another group of 31 women, in addition to receiving the same routine care as the first group, also participated in three classes of water aerobics per week. QOL was evaluated by applying the WHOQOL-BREF questionnaire in both groups at the 20^th^, 28^th ^and 36^th ^weeks of pregnancy. In the same occasions, women also answered another questionnaire about their experience with pregnancy and antenatal care.

**Results:**

The great majority of the participants considered that the practice of water aerobics had benefitted them in some way. QOL scores were found to be high in both groups during follow-up. There was no association between the practice of water aerobics and QOL.

**Conclusions:**

Further studies involving larger sample sizes should be conducted in different sociocultural contexts and/or using other instruments to adequately evaluate the QOL of women during pregnancy.

## Background

Pregnancy introduces physical and psychological changes into women's lives that may affect their individual perception of quality of life. The transformations women undergo during pregnancy may offer satisfaction and personal fulfillment; however, many women feel unattractive and heavy, and may also have difficulty with some movements and in performing routine activities [1,2]. The increase in body mass and its concentration in the upper body region result in greater mechanical load [3]. The burden on the spine, principally in the lumbar segment, affects posture, balance and locomotion.

The recommendations of the American College of Obstetricians and Gynecologists [4] and the Royal College of Obstetricians and Gynaecologists [5] state that pregnant women should be encouraged to engage in regular physical exercise programs of moderate intensity, a practice that has been shown to be safe and effective during pregnancy [6]. Various studies have indicated a series of benefits resulting from the practice of water aerobics by pregnant women such as, for example, reduced impact on articulations, less edema, increased diuresis [7], a significant reduction in arterial pressure [8], an increase in the volume of amniotic fluid [9], less need for analgesia [10], control of body weight [11], less back pain [12] and a reduction in postpartum depression [13]. In addition, psychological benefits such as improved well-being, satisfaction, self-confidence and body awareness have been reported [14,15]. Therefore, practice of this type of exercise may be one of the resources available to enable women to enjoy a good or better quality of life during pregnancy.

In Brazil, concern with the quality of life of pregnant women is one of the points emphasized in the Program for the Humanization of Prenatal Care and Childbirth launched in 2000. In this program, the Ministry of Health stipulates that, in addition to medical consultations, prenatal care should include counseling and information on how women should take care of their bodies during pregnancy, with a view to improving body awareness, mastering breathing relaxation techniques for better control at labor and childbirth and ensuring general well-being (Ministry of Health, 2001) [16]. However, there are few references in the literature on the role of either land or water-based physical exercise on women's quality of life during pregnancy [17-20]. This paper presents the findings of a study in which the association between the practice of water aerobics and quality of life was evaluated during pregnancy in a group of sedentary pregnant women attending prenatal care at a public healthcare service.

## Subjects and methods

A comparative study was carried out to evaluate the association between the practice of water aerobics and quality of life during pregnancy. The study was performed in conjunction with a controlled, randomized clinical trial, the objective of which was to evaluate the effectiveness and safety of a program of water aerobics of moderate intensity for sedentary pregnant women with respect to the outcome of the pregnancy, maternal weight gain, physical capacity and maternal cardiorespiratory parameters during labor and childbirth. Details on the intervention and the main results of this study are already published elsewhere [9-11].

Two groups of sedentary pregnant women with low-risk singleton pregnancies of ≤ 20 weeks, receiving prenatal care at a public healthcare service in Campinas, São Paulo, Brazil, who were expected to give birth at the Center for Women's Integrated Healthcare (CAISM) at the University of Campinas (UNICAMP), were evaluated. Exclusion criteria consisted of: women with a history of two or more Cesarean sections; women with neurological, cardiovascular, pulmonary, musculoskeletal or endocrine abnormalities confirmed by clinical and/or laboratory diagnosis; women with a body mass index (BMI) > 30; and those with any factor identified at the prenatal obstetrical evaluation as placing their health at risk.

Women who complied with the aforementioned criteria and agreed to participate in the study were immediately allocated to one of the two groups by means of a previously prepared, computer-generated randomization list. To guarantee the adequacy of this procedure, each number on the list corresponded to an op aque sealed envelope containing the questionnaires to be applied to each woman and the information referring to the intervention defined in the randomization procedure. Women in one group participated in a water aerobics program, while the women in the other group followed the prenatal care routinely provided at the clinic.

All the women were interviewed at the 20^th^, 28^th ^and 36^th ^weeks of pregnancy (± two weeks). On these occasions, two questionnaires were applied: one, specifically developed for this study, was used to evaluate the woman's perception of her prenatal care and the other to evaluate quality of life. The questionnaire selected to evaluate quality of life was the Portuguese language version of the WHOQOL-BREF [21], which was considered capable of measuring overall quality of life and was not limited to that related to the functional aspects of the health of pregnant women.

The women randomized to the water aerobics group participated in 50-minute water aerobics classes held three times weekly in an indoor pool heated to 28-30°C. The parameters followed were those recommended by the American College of Sports Medicine [22], which proposes 3-5 classes a week, a training zone of 55-65% of maximum heart rate, 20-60 minute classes, maximum heart rate of 140 bpm and maintaining body temperature below 38°C.

In the study group that participated in the water aerobic classes, 31 women were evaluated at the time of admission to the study, 23 women at the 28-week follow-up and 20 women at the follow-up visit in the 36^th ^week of pregnancy. In the control group, 35 women were evaluated at admission, 26 at 28 weeks and 23 at 36 weeks of pregnancy.

To compare the quality of life scores in the two study groups, multivariate analysis of variance (MANOVA) was used for repeated measures or Friedman test when data distribution was not normal. This analysis also evaluated the variation in quality of life scores at the different assessment moments: at baseline (20^th ^week), at the 28^th ^week and at the 36^th ^week of pregnancy. Statistical analysis was performed using the SAS software program, version 8.2 and statistical significance was defined as p < 0.05.

The study protocol was approved by the Institutional Review Board of the School of Medical Sciences, University of Campinas (UNICAMP) on May 20, 2003 under reference #080/2003. The women participated voluntarily and signed an informed consent form. The confidentiality of all data was respected in conformity with Resolution 196/96 [23] of the Brazilian Ministry of Health's National Health Council on Research involving Human Beings.

## Results

The mean age of the women who participated in the water aerobics classes was 26 years compared to a mean of 24 years in the control group. Most of the women had eight or more years of schooling: 52% of those in the water aerobics group and 83% of those in the control group, this difference being statistically significant (p = 0.0065). The majority of the husbands or partners of these women also had more than elementary schooling, 70% in the water aerobics group and 62% in the control group, and there was no statistically significant difference between the two groups in this respect. The mean family income of the women in the water aerobics group was US$477.10 ± US$712 and US$402.56 ± US$859 in the control group. Most of the women in the two groups were unemployed when they were admitted to the study: 55% in the water aerobics group and 63% in the control group. Of those who were employed, the majority in both groups worked full-time day shifts as domestic servants, salespersons or cashiers. At the time of admission to the study, there was no statistically significant difference between the women in the two groups with respect to the practice of physical exercise, 58% in the water aerobics group and 63% in the control group reporting having practiced some form of physical exercise in the past. Of those who reported a history of having practiced some form of physical exercise, most reported practicing sports in general at school (elementary or high school) and more than one-third had at some time joined a gym (data not presented as tables). At any rate, none of the women were currently practicing any form of routine, regular physical activity.

Most of the women in the water aerobics group, 71% at admission to the study and 56% at 28 weeks of pregnancy, expected the exercise to improve their physical well-being. At the end of the study, the majority of women (65%) believed that having participated in the water aerobics classes would make childbirth easier for them and 25% continued to believe that it had been good for their physical well-being. The great majority, around 90%, of the participants interviewed at 28 and 36 weeks of pregnancy considered that the practice of water aerobics had benefitted them in some way. The majority of women who attended the 28 and 36-week follow-up visits (74% and 60%, respectively) reported that it had been no problem to attend the classes (Table 1).

**Table 1:** Expectations of the women in the water aerobics group, their perception of the benefits obtained and the difficulties found in attending classes, according to evaluations performed at 28 and 36 weeks of pregnancy.

	**Baseline**	**28 Weeks**	**36 Weeks**
	
**Expectations**			
Improve physical well-being	71	56	25
Improve the baby's physical well-being	19	4	10
Make childbirth easier	19	30	65
Help keep in shape/control weight	19	-	-
			
**Perception of benefits**	-	91	90
			
**No difficulty in attending classes**	-	74	60
			
**Total number of women**	31	23	20

Data expressed as percentages.

Slightly less than half the women in the water aerobics group (45.2%) and 77% of the control group stated that they were happy/very happy when they learned that they were pregnant; however, almost all the women of both groups (96.8% and 97.1%, respectively) stated that they were happy/very happy with their pregnancy at the time of admission to the study (data not presented as tables).

**Figure 1:**
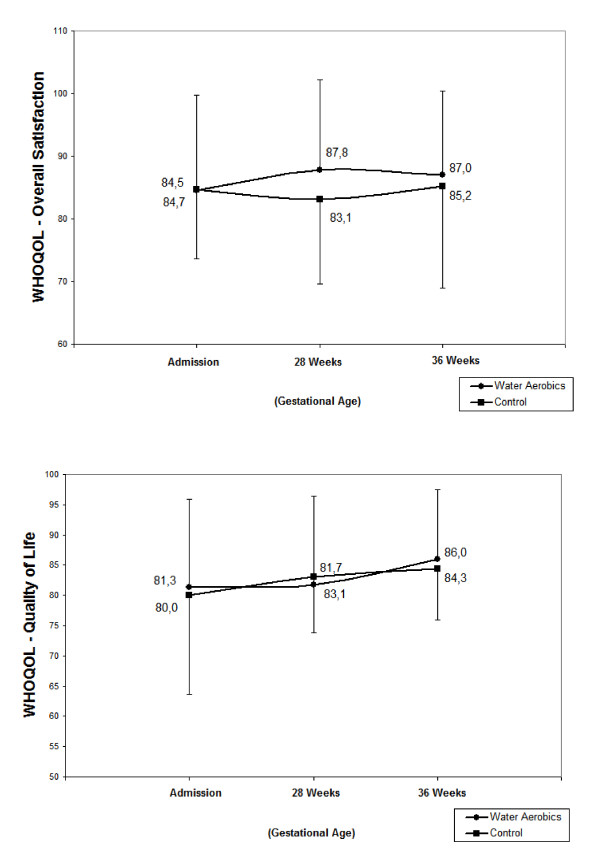
**Mean scores of overall satisfaction and quality of life**.

**Figure 2:**
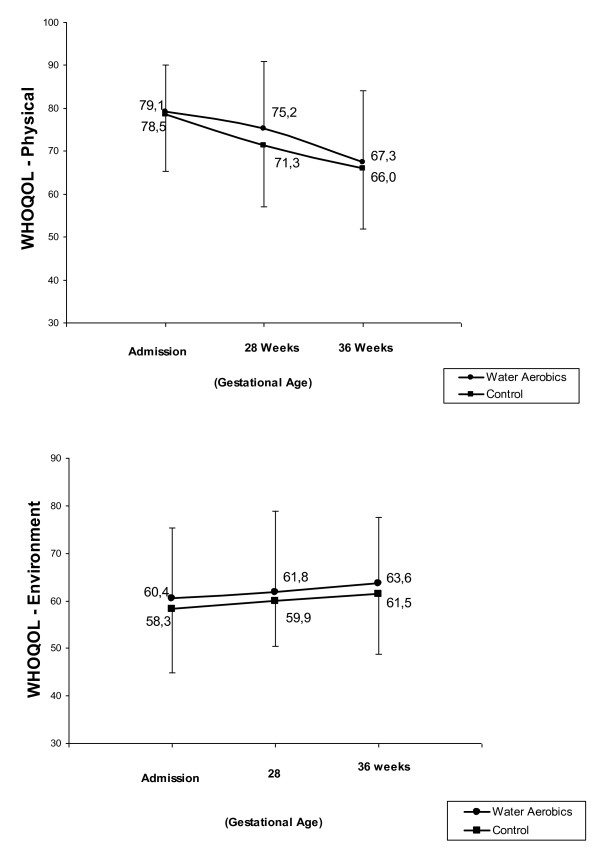
**Mean scores of physical and environment domain**.

The mean overall scores of quality of life and satisfaction of the women in the two groups were high at all the evaluation moments, around 80 points (Figure 1). In the different domains evaluated by the WHOQOL-BREF, a similar overall situation was found, with means of around 70 points. The exception referred to the environment domain in which mean scores were close to 60 points. In this domain, mean scores increased significantly over time, rising from 60.4 points in the water aerobics group and 58.3 points in the control group at admission to 63.6 and 61.5 points, respectively, at 36 weeks of pregnancy. On the other hand, in the physical domain, a significant decrease was found in mean quality of life scores over time, decreasing from a mean of 79.1 points in the water aerobics group and 78.5 points in the control group at admission to the study to 67.3 and 66.0 points, respectively, at 36 weeks of pregnancy (Figure 2). However, no statistically significant differences were found between the groups in any of the domains. Multiple analyses failed to detect any variables associated with the scores in the different domains.

## Discussion

The present findings show that, in general, the two groups of women studied scored highly in the different domains used to evaluate quality of life during pregnancy. This may be surprising in a group of women obliged to resort to the public healthcare system in a developing country. Even in the environment domain in which scores were lowest right from the beginning of the study, there was positive progress over time. On the other hand, there was no association between the practice of water aerobics and quality of life during pregnancy.

First, it must be taken into consideration that quality of life is a subjective concept, the evaluation of which is relatively complex and difficult to measure [24]. Based on the definition of quality of life adopted by the World Health Organization [25], the complexity surrounding the construction of this concept for the individuals themselves is perceptible, since it deals with an interrelationship between the environment and individual physical and psychological aspects, independence level, social relationships and personal beliefs. In addition, this interrelationship exists within a certain cultural context, within the context of the system of values in which each individual lives and in relation to their objectives, concerns, expectations and standards [26]. This means that any measurement of quality of life ideally needs to achieve an accurate translation of this set of elements into an index or score that reflects the perception of different individuals at different circumstances of their lives on how their life actually is [27]. Therefore, judging by the scores recorded at admission to this study, the women interviewed in the two groups were satisfied with their insertion in their sociocultural environment and with respect to the objectives and expectations they nurtured, including their pregnancy. It should be emphasized that the overall satisfaction score recorded at the beginning of the study was around 84 points in both groups and at the end of the study 87 points in the water aerobics group and 85 points in the control group, indicating that these women's perception of a good quality of life remained stable. These findings are compatible with the results reported by Tendais et al. [28] in Portugal in a study in which a group of pregnant women had higher overall quality of life scores compared to a group of non-pregnant women. On the other hand, Dresher et al. [29] found a slight variation in quality of life scores in pregnant teenagers.

Particularly with respect to the environment domain, it is reasonable to believe that the fact that the participants of the study were receiving prenatal care at a teaching hospital, the quality of whose services is recognized throughout the region, may have contributed to increasing their score over time. This fact may be explained by the fact that this domain is evaluated on the basis of answers to questions regarding not only physical safety and protection, the home environment, financial resources, participation in and opportunities for recreational and leisure activities, physical environment (pollution/noise/traffic/climate) and transportation, but also with respect to healthcare and social assistance (availability and quality) and opportunities for acquiring new information and skills.

The homogeneity between the groups with respect to the women's perception of their quality of life will certainly have contributed to the fact that no statistically significant difference in scores was found between the groups throughout the study period, although in some cases there was a variation over time. It was expected that at least in the physical domain an association would be found between the practice of exercise and quality of life, particularly because the findings of the original clinical trial [9-11] indicated the safety of these activities for women and their babies as well as additional benefits that included a reduced need for analgesia during delivery and increased amniotic fluid following immersion. In addition, at 28 and 36 weeks of pregnancy the women who had attended the water aerobics classes reported that this form of exercise improved their physical well-being. Nevertheless, comparing the quality of life scores in this domain, no statistically significant difference was found between the two groups: scores tended to decrease in both groups as the pregnancy progressed, which is unsurprising bearing in mind the effect of pregnancy on the female body.

The lack of any association between the practice of physical exercise and quality of life scores may also be attributed to the small number of cases included in this study. On the other hand, it must be remembered that these women already had a high perception of their quality of life at admission to the study, which was fundamental for the benefits of the practice of physical activity not to have a significant repercussion on the other domains evaluated. Another possibility that should also be taken into consideration is that the instrument used to measure quality of life may not have been the most sensitive tool for the detection of changes during pregnancy. To the best of our knowledge, there is no reference in the literature to the use of the WHOQOL-BREF for measuring quality of life during pregnancy, although it has been validated in several different contexts with the intention that it would serve as a useful instrument for application in a wide range of situations [21]. On the other hand, there is a known scarcity of specific instruments for the evaluation of quality of life in pregnant women and recent mothers. At the same time, given the nature of the process of pregnancy, it must be taken into consideration that any instrument proposed to evaluate the quality of life of pregnant women should focus on their well-being and not only on clinical indicators [30]. Therefore, the WHOQOL-BREF was expected to represent a useful tool for the evaluation of the quality of life of the women participating in the present study.

It should be emphasized, however, that the results presented here cannot be used as arguments to discourage the practice of water aerobics during pregnancy as a means of improving women's well-being and quality of life. On the contrary, these findings should motivate further studies to be carried out with larger sample sizes and in different contexts. In addition, other instruments should be used to evaluate quality of life.

## Conclusion

There was no association between the practice of physical exercise and quality of life during pregnancy. Further studies involving larger sample sizes should be conducted in different sociocultural contexts and/or using other instruments to adequately evaluate the QOL of women during pregnancy.

## Abbreviations

QOL: Quality of Life; WHOQOL-BREF: World Health Organization Quality of Life abbreviated questionnaire; ACOG: American College of Obstetricians and Gynecologists; RCOG: Royal College of Obstetricians and Gynaecologists; CAISM: Center for Women's Integrated Healthcare; UNICAMP: University of Campinas; BMI: body mass index; MANOVA: multivariate analysis of variance; SAS: statistical and predictive analysis software.

## Competing interests

The authors declare that they have no competing interests.

## Authors' contributions

ALBAV, MJDO and JGC participated in all steps of the study, including research planning, data collection, analysis and writing the manuscript. EPB, CS and SRC participated in data collection and review of the manuscript. All authors gave suggestions, read the manuscript carefully, fully agreed on its content and approved its final version.
